# Mental health and psychosocial function of general population during the COVID‐19 epidemic in China

**DOI:** 10.1002/ctm2.103

**Published:** 2020-06-11

**Authors:** Qi Zhou, Zhenyu Hu, Guolin Bian, Haihang Yu, Xingxing Li, Yin Lu, Chang Yu, Xianglan Li, Qin Yao, Wenhua Zhou, Ti‐Fei Yuan, Dongsheng Zhou

**Affiliations:** ^1^ Ningbo Key Laboratory of Sleep Medicine Ningbo Kangning Hospital Ningbo Zhejiang China; ^2^ Laboratory of Behavioral Neuroscience, Ningbo Addiction Research and Treatment Center, School of Medicine Ningbo University Ningbo China; ^3^ Shanghai Key Laboratory of Psychotic Disorders, Shanghai Mental Health Center Shanghai Jiao Tong University School of Medicine Shanghai China; ^4^ Co‐innovation Center of Neuroregeneration Nantong University Nantong China

Dear Editor,

Since December 2019, COVID‐19 caused by severe acute respiratory syndrome coronavirus 2 (SARS‐CoV‐2) emerged and rapidly spread to many countries.^1^, ^2^ As of 10 April 2020, the number of confirmed cases in China^3^ has increased to 83 307 and confirmed cases in the world have increased to 1 439 516.^4^ The COVID‐19 epidemic has resulted in serious threats to health and safety, as well as various psychological problems, such as high levels of perceived stress, insomnia, anxiety, and depression symptoms.^5^ Previous studies have reported that the medical workers experiencing tremendous pressure, from factors, such as high risk of infection, overwork, depression, and emotionally impacted by negative patients, and lack of protection for their families, have been shown to exhibit long‐term psychological implications.^6^, ^7^ This study utilized an online survey method to measure the incidence and severity of psychological disturbances in the general public.

We conducted a cross‐sectional study through an online survey from 23 February 2020 to 1 March 2020. Using the Questionnaire Star platform, we provided the QR code of the questionnaire survey online for general public nationwide. This investigation period corresponded to the decreasing period after the maximum point of COVID‐19 outbreak in China, 2 months after the COVID‐19 epidemic outbreak in Wuhan. All the subjects who participated in the survey had asked whether they would like to participate in the survey prior to their enrollment. The online survey includes questions about sociodemographic and clinical variables. In addition, Self‐Reporting Questionnaire‐20 (SRQ‐20), Athens Insomnia Scale (AIS), and Chinese version of the Perceived Stress Scale (CPSS) were used to evaluate the stress responses (i.e., ≥7 in SRQ‐20), insomnia (i.e., ≥6 in AIS), and stress (i.e., ≥29 in CPSS).

Among all the respondents, 784 were male (32.2%) and 1651 were female (67.8%). We did not observe any gender differences in the SRQ‐20 and AIS scales. The perceived stress (CPSS score) was higher in the male than the female (27.26 vs. 27.03; *P* = .009). People younger than 20 years had the lowest AIS scores (mean [SD] = 2.85 [3.31]) and CPSS scores (mean [SD] = 26.47 [9.48]). Those above 60 years old showed the highest score on AIS scores (mean [SD] = 4.02 [4.80]) and CPSS scores (mean [SD] = 30.09 [10.8]). Divorced individuals showed the highest score on AIS scores (mean [SD] = 3.57 [4.51]) and CPSS scores (mean [SD] = 27.62 [8.93]). Out of 14 different occupations, farmers scored the highest on AIS scores (mean [SD] = 6.56 [7.99]) and the civil servants had the highest score on CPSS scores (mean [SD] = 30.59 [8.54]). Participants with low literacy had the highest AIS scores (mean [SD] = 10.67 [12.22]) and CPSS scores (mean [SD] = 31.00 [28.48]). In comparison to other provinces, Hubei province presented the highest AIS scores (mean [SD] = 4.63 [8.05]) and CPSS scores (mean [SD] = 32.63 [12.27]) (Table 1 and Figure 1).

**TABLE 1:** Sociodemographic characteristics in general population

	N (%)	SRQ	AIS	CPSS
Gender				
Male	784 (32.2)	1.97 ± 3.36	3.26 ± 3.53	27.26 ± 10.09
Female	1651 (67.8)	1.76 ± 2.89	3.11 ± 3.30	27.03 ± 8.03
*P*‐value	‐	.805	.766	.009**
Age (years)				
<20	377 (15.48)	1.73 ± 2.99	2.85 ± 3.31	26.47 ± 9.48
20‐39	1505 (61.81)	1.73 ± 2.97	3.11 ± 3.32	26.85 ± 8.62
40‐59	510 (20.94)	2.16 ± 3.24	3.46 ± 3.43	28.07 ± 8.24
≥60	43 (1.77)	2.07 ± 3.73	4.02 ± 4.80	30.09 ± 10.82
*P*‐value	‐	.194	.007**	<.001**
Marital status				
Married	1114 (45.75)	1.92 ± 3.02	3.50 ± 3.46	27.61 ± 8.66
Unmarried	1273 (52.28)	1.71 ± 3.03	2.86 ± 3.24	26.67 ± 8.76
Widowed	6 (0.25)	1.67 ± 2.07	2.00 ± 2.10	24.17 ± 16.02
Divorced	42 (1.72)	2.83 ± 4.37	3.57 ± 4.51	27.62 ± 8.93
*P*‐value	‐	.205	<.001**	<.001**
Occupation				
Farmer	9 (0.37)	6.00 ± 7.33	6.56 ± 7.99	29.89 ± 19.10
Enterprise staff	225 (9.24)	1.68 ± 2.66	3.03 ± 3.16	28.02 ± 8.83
Business unit personnel	214 (8.79)	1.98 ± 3.27	3.24 ± 3.09	28.60 ± 8.71
Self‐employed businessman	18 (0.74)	2.61 ± 3.74	3.72 ± 3.23	25.61 ± 10.63
Soldier	1 (0.04)	NA	NA	NA
Student	1069 (43.9)	1.66 ± 2.93	2.77 ± 3.18	26.47 ± 8.88
Immigrant laborer	5 (0.21)	4.60 ± 4.22	5.00 ± 3.16	27.40 ± 4.39
Medical staff	729 (29.94)	1.90 ± 3.15	3.51 ± 3.55	27.07 ± 8.21
Civil servant	27 (1.11)	2.41 ± 2.58	4.56 ± 4.33	30.59 ± 8.54
Freelance	24 (0.99)	2.67 ± 3.17	3.71 ± 3.53	28.00 ± 8.48
Retiree	41 (1.68)	1.59 ± 2.43	3.51 ± 3.65	29.83 ± 8.72
Unemployed	11 (0.45)	3.27 ± 3.80	4.00 ± 3.22	23.73 ± 9.01
Others	62 (2.54)	1.96 ± 2.92	3.89 ± 3.43	27.14 ± 8.93
*P*‐value	‐	.578	<.001**	<.001**
Education level				
Illiteracy	3 (0.12)	6.67 ± 11.55	10.67 ± 12.22	31.00 ± 28.48
Primary	5 (0.21)	6.60 ± 8.11	5.00 ± 3.81	22.60 ± 14.55
Junior middle school	38 (1.56)	2.58 ± 3.79	3.39 ± 3.65	28.95 ± 11.28
High school	85 (3.49)	1.95 ± 2.65	3.49 ± 3.31	25.72 ± 10.68
College	1202 (49.36)	1.71 ± 2.99	2.87 ± 3.26	26.48 ± 8.95
Undergraduate	771 (31.66)	1.82 ± 2.92	3.38 ± 3.36	27.60 ± 7.91
Bachelor degree or above	331 (13.59)	2.03 ± 3.25	3.50 ± 3.55	28.40 ± 8.42
*P*‐value	‐	.580	<.001**	<.001**
Geographical location				
Hubei province	8 (0.33)	3.38 ± 6.82	4.63 ± 8.05	32.63 ± 12.27
Zhejiang province	1161 (47.68)	1.86 ± 2.98	3.40 ± 3.42	27.52 ± 8.40
Guangdong province	9 (0.37)	1.11 ± 2.67	1.33 ± 1.73	22.33 ± 10.54
Henan province	21 (0.86)	3.67 ± 5.53	4.19 ± 4.35	30.05 ± 7.04
Other regions of the country	1232 (50.60)	1.77 ± 3.02	2.92 ± 3.26	26.68 ± 8.98
Abroad	4 (0.16)	0.00 ± 0.00	2.75 ± 3.59	21.25 ± 16.46
*P*‐value	‐	.495	<.001**	.003**

*Note*. Data are presented as mean ± SD. Significant results are highlighted (*P* < .05) in bold. Mann‐Whitney U test or Kruskal‐Wallis test for independent samples.

Abbreviations: SRQ‐20, Self‐Report Questionnaire‐20; AIS, Athens Insomnia Scale; CPSS, Chinese version of the Perceived Stress Scale; NA, not applicable.

**FIGURE 1:**
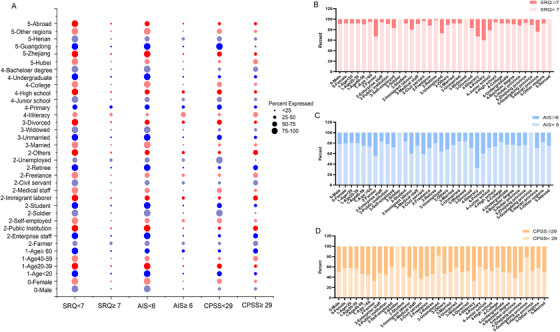
Distribution of different populations above and below the critical value of different scales. A, The scores of different populations and different scales were compared. The size of the bubble represents the proportion of different people in a scale. B, The scores of different populations and the Self‐Report Questionnaire‐20 Scale (SRQ‐20) were compared. C, The scores of different populations and the Athens Insomnia Scale (AIS) were compared. D, The scores of different populations and the Chinese version of the Perceived Stress Scale (CPSS) were compared

Spearman's correlation analysis (Table S1) showed that there were significant correlations between SRQ‐20, AIS, and CPSS scales with age, marital status, and geographical location. A binary logistic regression analysis (Table S2) showed that the risk of insomnia in the general population was independently associated with four variables: age, occupation, marital status, and geographical location (*P* < .05). The risk of stress in the general population was independently associated with three variables: gender, occupation, and education level (*P* < .05). No risk factors associated with stress response were found in the general population. Further multiple step‐wise regression analyses (Table S3) showed that age was associated with SRQ‐20 scores; occupation and marital status were associated with AIS scores; and age and education level were associated with CPSS scores.

The present study investigated the prevalence of psychosocial issues in the general population in the COVID‐19 epidemic environment. Age, education, and profession were shown to be significant risk factors contributing to the stress vulnerability for the general population. Our report found people older than 60 years reported more severe symptoms on all scales. One possibility is that the elderly were found to have the highest mortality rates during the COVID‐19 epidemic. Another possibility is that older adults were unable to go out and engage in normal social interactions. Therefore, this may have caused greater psychological stress. Another particularly affected group was farmers, who may have concerns regarding inadequate medical skills and conditions in rural areas. It is noteworthy that people with lower levels of education experienced the highest level of distress among all occupations, which may be due to insufficient knowledge and reputable sources of information. The concerns about whether they can return to work, extended working hours, and reduced expected income may explain the high stress level among people of different occupations forced to suspend work and production during the epidemic. Meanwhile, compared with areas outside Hubei Province, people in Hubei Province reported more severe symptoms of insomnia and stress, which is likely due to the fact that Hubei Province was the origin and center of the epidemic.

We also found that the degree of public psychological distress decreased significantly with time, and some of the measurement scales did not reach clinical levels of severity. Two major reasons may explain the low psychological stress response in general population. First, during the COVID‐19 epidemic, both local and national mental health institutions have been providing psychological counseling or intervention through various means, including telephone, Internet, and social media applications.^8^ Second, the Chinese government took sweeping measures to ensure public education, medical security, medical isolation, control of population mobility, and resource allocation across the country.^9^ Moreover, measures were undertaken to strengthen individual protection, reduce gatherings, and reduce outings to stop the spread of the virus, and the number of new cases has been gradually brought under control.

In conclusion, we found that the prevalence of psychosocial symptoms and risk factors was higher in vulnerable groups such as the elderly, farmers, and individuals with low literacy levels. In order to reduce psychosocial distress and prevent further mental health problems, it will be necessary to institute a proper mental health and well‐being system targeted to the public.

## CONFLICT OF INTEREST

The authors declare no conflict of interest.

## Supporting information

Supporting Material
