# Supplementary material for: Mental health and psychosocial function of general population during the COVID‐19 epidemic in China
Source: Clin Transl Med. 2020 Jun 11;10(2):e103. doi: 10.1002/ctm2.103 (PMC7300737; doi:10.1002/ctm2.103)
Supplement: Supplementary file 1 — Supporting Material [file CTM2-10-e103-s001.doc]

**Table S1. Spearman Correlations between Sociodemographic Characteristics with Scales**

|  | **Mean** | **SD** | **Gender** | **Age** | **Marital status** | **Occupation** | **Education level** | **Geographical location** | **SRQ** | **AIS** | **CPSS** |
| --- | --- | --- | --- | --- | --- | --- | --- | --- | --- | --- | --- |
| **Gender** | 1.678 | 0.467 | 1 |  |  |  |  |  |  |  |  |
| **Age** | 2.090 | 0.653 | **-0.082**** | 1 |  |  |  |  |  |  |  |
| **Marital status** | 1.579 | 0.594 | **0.041*** | **-0.532**** | 1 |  |  |  |  |  |  |
| **Occupation** | 6.265 | 2.375 | **0.124*** | **0.145**** | **-0.183**** | 1 |  |  |  |  |  |
| **Education level** | 6.510 | 0.864 | -0.014 | **0.242**** | **0.232**** | **-0.500**** | 1 |  |  |  |  |
| **Geographical location** | 3.542 | 1.498 | -0.032 | **-0.466**** | **-0.298**** | **0.609**** | **-0.539**** | 1 |  |  |  |
| **SRQ** | 1.827 | 3.051 | -0.005 | **0.070**** | **-0.062**** | 0.030 | 0.029 | **-0.047*** | 1 |  |  |
| **AIS** | 3.161 | 3.377 | -0.006 | **0.063**** | **-0.095**** | **0.060**** | **0.066**** | **-0.076**** | **0.580**** | 1 |  |
| **CPSS** | 27.106 | 8.744 | **-0.053**** | **0.097**** | **-0.094**** | -0.018 | **0.102**** | **-0.076**** | **0.215**** | **0.189**** | 1 |

SRQ-20: Self-Report Questionnaire-20; AIS: Athens Insomnia Scale; CPSS: Chinese version of the Perceived Stress Scale; Significant results are highlighted (*p*<0.05) in bold.

**Table S2.** Risk Factors for Mental Health Outcomes Identified by Multivariable Logistic Regression Analysis

|  | **Unadjusted OR (95% CI)** | | | | | |
| --- | --- | --- | --- | --- | --- | --- |
|  | **SRQ** | ***p* value** | **AIS** | ***p* value** | **CPSS** | ***p* value** |
| **Gender** | 0.756(0.538-1.061) | 0.105 | 0.822(0.653-1.034) | 0.095 | 0.429(0.242-0.761) | **0.004** |
| **Age** | 1.039(0.809-1.335) | 0.765 | 1.292(1.094-1.526) | **0.003** | 0.984(0.635-1.525) | 0.944 |
| **Marital status** | 1.080(0.823-1.416) | 0.580 | 0.792(0.652-0.962) | **0.019** | 1.333(0.857-2.071) | 0.202 |
| **Occupation** | 1.027(0.959-1.101) | 0.445 | 1.065(1.016-1.115) | **0.008** | 0.885(0.785-0.997) | **0.044** |
| **Education level** | 0.995(0.823-1.203) | 0.959 | 1.014(0.892-1.151) | 0.835 | 0.704(0.523-0.948) | **0.021** |
| **Geographical location** | 0.996(0.892-1.111) | 0.938 | 0.881(0.819-0.949) | **0.001** | 1.069(0.882-1.295) | 0.498 |

SRQ-20: Self-Report Questionnaire-20; AIS: Athens Insomnia Scale; CPSS: Chinese version of the Perceived Stress Scale; OR, odds ratio; Significant results are highlighted (*p*<0.05) in bold.

**Table S3.** Predictors generated by Multivariate Logistic Regression with scale scores as dependent variables.

|  | **Coefficients** | | | | | | | | **95.0% Confidence Interval for B** | | | | |  |
| --- | --- | --- | --- | --- | --- | --- | --- | --- | --- | --- | --- | --- | --- | --- |
|  | **B** | | **Std.Error** | | **T** | | ***p* value** | | | **Lower Bound** | | **Upper Bound** | |  |
| **SRQ** |  | |  | |  | |  | | |  | |  | |  |
| **(Constant)** | | 1.371 | | 0.207 | | 6.620 | | **<0.001***** | | | 0.965 | | 1.777 | |
| **Age** | | 0.218 | | 0.095 | | 2.308 | | **0.021** | | | 0.033 | | 0.404 | |
| **AIS** | |  | |  | |  | |  | | |  | |  | |
| **(Constant)** | | 3.403 | | 0.268 | | 12.703 | | **<0.001***** | | | 2.878 | | 3.928 | |
| **Occupation** | | 0.063 | | 0.029 | | 2.186 | | **0.029** | | | 0.006 | | 0.119 | |
| **Marital status** | -0.402 | | 0.115 | | -3.499 | | **<0.001***** | | | -0.628 | | -0.177 | |  |
| **CPSS** |  | |  | |  | |  | | |  | |  | |  |
| **(Constant)** | 21.452 | | 1.402 | | 15.303 | | **<0.001***** | | | 18.704 | | 24.199 | |  |
| **Age** | 0.854 | | 0.272 | | 3.138 | | **0.002**** | | | 0.321 | | 1.388 | |  |
| **Education level** | 0.594 | | 0.206 | | 2.888 | | **0.004**** | | | 0.191 | | 0.998 | |  |
